# 11th Tuscany Retreat on Cancer Research and Apoptosis: Genetic profiling, resistance mechanisms and novel treatment concepts in cancer and neurodegeneration

**DOI:** 10.1038/s41419-019-2166-0

**Published:** 2019-12-05

**Authors:** Justin Pogmore, Joseph Longo, Diana Resetca, Negin Farivar, Niall A. Buckley, María G. Rincón, Christina M. Bebber, Anusha Venkatraman, James M. Pemberton

**Affiliations:** 1 Biological Sciences, Sunnybrook Research Institute, Toronto, ON M4N 3M5 Canada; 2 Department of Biochemistry, University of Toronto, Toronto, ON M5S 2J7 Canada; 3 Princess Margaret Cancer Centre, University Health Network, Toronto, ON M5G 1L7 Canada; 4 Department of Medical Biophysics, University of Toronto, Toronto, ON M5S 2J7 Canada; 5 Vancouver Prostate Centre, Vancouver, BC V6H 3Z6 Canada; 6 Department of Experimental Medicine, University of British Columbia, Vancouver, BC V5Z 1M9 Canada; 7 Medical Research Council (MRC) Toxicology Unit, University of Cambridge, Cambridge, UK CB2 1QP; 8 Institute of Molecular Medicine and Cell Research, Faculty of Medicine, Albert Ludwigs University Freiburg, Stefan Meier Strasse 17, 79104 Freiburg, Germany; 9 Faculty of Biology, Albert Ludwigs University Freiburg, Schänzlestrasse 1, 79104 Freiburg, Germany; 10 Spemann Graduate School of Biology and Medicine (SGBM), Albert Ludwigs University Freiburg, Albertstrasse 19a, 79104 Freiburg, Germany; 11 Department of Translational Genomics, Medical Faculty, University of Cologne, Weyertal 115b, 50931 Cologne, Germany; 12 Cologne Excellence Cluster on Cellular Stress Response in Aging-Associated Diseases (CECAD), Medical Faculty, University of Cologne, Joseph-Stelzmann-Straße 26, 50931 Cologne, Germany; 13 Department I of Internal Medicine, University Hospital of Cologne, Kerpener Str. 62, 50937 Cologne, Germany

**Keywords:** Cell death, Cancer models

For the 11th time, an international assembly of scientists gathered in the medieval castle of Palazzo di Piero, near the town of Chiusi, Italy, to exchange ideas and present their research. The opening presentation was given by Brent Derry (SickKids, Canada), who showed that alternative polyadenylation regulates oncogenic Ras in both *C. elegans* and human cancer cells. His student, Matthew Eroglu, showcased that genes *mstr-1* and *mstr-2* are essential for cells to make proper fate decisions. Guy Taichman, student of Oded Rechavi (Tel Aviv University, Israel), showed that stress causes a resetting of heritable RNAi in *C. elegans*.

Ivano Amelio (University of Cambridge, UK) identified that mutant p53 interacts with HIF1α and alters chromatin accessibility/architecture. Emanuele Panatta of the Melino group generated a p73 C-terminal domain mutant mouse demonstrating that this region is necessary for proper neurological developmental. Niall Buckley, student of Dr. Melino, showed that p73 has important function in the development of epithelial cell cilia, while María Rincón, student of Christoph Borner, reported that lung epithelial cells treated with Gliotoxin and GSK-3 inhibitors display inhibited caspase-3 activation and high LDH release.

Silvia von Karstedt (University of Cologne, Germany) showed that ferroptosis may represent a means of selection in human cancers. Dr. von Karstedt’s students then followed; Christina Bebber highlighted that responsiveness to ferroptosis is dependent on ASCL-1. Fabienne Müller showcased MEFs models harboring different KRAS point mutations exhibit differential ferroptosis sensitivities. Moreover, Laura Prieto-Clemente demonstrated a novel role of mitochondria in regulating ferroptosis.

Revuen Stein (Tel Aviv University, Israel) proposed to target CAFs in glioma by using an inhibitor of CD38. His students, Or Ganon and Kaveri Banerjee, presented data to suggest an unusual distribution of microglia and macrophages in a mouse model of glioma. Barak Rotblat (Ben-Gurion University of the Negev, Israel) presented his research on newly identified cancer cell vulnerabilities under glucose starvation. His student, Tal Levy, demonstrated that glioblastoma cells under glucose starvation highly depend on 4EBP1, a repressor of protein translation.

Ronit Pinkas-Kramarski (Tel Aviv University, Israel) and her student Rawan Bassal presented their research on the role of autophagy in Alzheimer’s disease and neurodegeneration. Other trainees in the Pinkas-Kramarski group (Hanan Abu Taha, Eya Wolfson, and Adva Kochavi) showcased their work on the ErbB family of receptors in cancer, with a focus on disrupting the crosstalk between ErbB, Ras and Nucleolin with small molecule inhibitors.

Hartmut Juhl (Indivumed, Germany), introduced the Indivutype platform, which combines genomic, transcriptomic, microRNA and proteomic datasets from high-quality cancer biospecimens. John Babcook (Zymeworks, Canada) introduced the novel Azymetric™ platform for the development of IgG-like bispecific antibodies for the targeting of synergistic drug targets.

Gerry Melino (University of Cambridge, UK) showcased the dynamic modulation of expression of the ZNF281 zinc finger protein at the DNA damage site during genotoxic stress. Massimiliano Agostini (University of Tor Vergata, Italy) presented the role of the transcription factor ZNF750 in terminal differentiation of the epidermis. His student, Consuelo Pitolli, presented on the role of the transcription factor ZNF281 in neuroblastoma. Lukas Peintner presented his work on the regulation of DNA damage response in monogenic polycystic kidney disease, proving that loss of polycystin-1 severely impacts Bcl2 family regulation and DNA repair.

Anne Hamacher-Brady (Johns Hopkins University, USA) described that during BAX-mediated MOMP, mitochondria are targeted by endolysosomal vesicles, and that these inter-organelle interactions are fundamental to BAX pore formation in cells. Liora Lindenboim demonstrated that Bax interacts with the nuclear cytoskeleton via interaction with Nesprin proteins and the LINC complex. David Andrews (Sunnybrook Research Institute, Canada) explained how AI analysis of microscopy data can be used to evaluate apoptosis and other cell responses, emphasizing potential utility in precision medicine. His student, Justin Pogmore, presented his work on pharmacologically targeting Bax, while James Pemberton suggested that the mechanism of Puma could explain how neurons commit to either death or axon degeneration. Christoph Borner (University of Freiburg, Germany), showcased a novel interaction between Hexokinase-1 and Puma.

Linda Penn (University Health Network, Canada) and her student Diana Resetca presented the use of MYC-BioID technology for the identification of actionable protein-protein interactions of the MYC family of oncoproteins. Joseph Longo, student of Dr. Penn, demonstrated how the statin family of cholesterol-lowering drugs could be used to induce apoptosis in a specific subset of high-risk multiple myeloma.

Mads Daugaard (Vancouver Prostate Centre, Canada) showed that cisplatin-resistance in bladder cancer can be dissected via genome-wide CRISPR screening and targeted by VAR2-drug conjugates. Chris Kedong Wang showed that cancer exosomes contain high levels of chondroitin sulfate proteoglycan. Nastaran Khazamipour discussed the progress toward SPY CAR-T cell engineering for elimination of tumor cells, while Negin Farivar reported on engineering a plasmonic photothermal therapy to ‘zap’ tumor cells in the blood circulation using gold nanorods. Nader Al-Nakouzi spoke about the investigation of hormone-dependent regulation of glycosaminoglycan signaling in prostate cancer.

Ulrich Maurer (University of Freiburg, Germany) reported effects of SPATA2 knockout on immune cells in mice. His student, Laura Griewahn, elucidated the role of SPATA2 in intestinal cells. Mads Gyrd-Hansen (University of Oxford, UK) showed how SPATA2 facilitates CYLD recruitment to TNFR. Thomas Brunner (University of Konstanz, Germany) showed that colorectal tumors can synthesize and release immunosuppressive glucocorticoids, suppressing the anti-tumor immune response. Michael Bergmann (Medical University of Vienna, Austria) presented data indicating that radiotherapy polarizes tumor-associated macrophages from the M2 towards an M1-like phenotype. Johannes Längle (Bergmann group) found an impact of proteins associated with DNA damage, ER stress response, immunogenic cell death and stress granules on overall survival in liver metastases. Sebastian Carotta (Boehringer Ingelheim, Vienna) presented the mechanism of the STING pathway in regulating immune and cancer cell death. Molecular oncology work presented by Mahvash Tavassoli (King’s College, UK) focused on EGFR dynamics and radiotherapy response in head and neck cancer, and its propagation by HPV infection. Anusha Venkatraman, Student of Christoph Borner, showcased a novel virally-encoded miRNA rendering cells resistant to genotoxic stress-induced apoptosis. Maria Sibilia (Medical University of Vienna, Austria) indicated that targeting EGFR on non-cancerous cells in the tumor microenvironment results in reduced cancer development.

Erwin Wagner (Medical University Vienna, Austria) showed how the dimeric transcription factor AP-1 plays a role in inflammatory skin disease such as psoriasis, atopic dermatitis, and inflammation-associated liver cancer. John Silke (WEHI, Australia) showcased mutations that prevent cleavage of RIPK1 by Caspase-8 induce inflammation in humans and mice.

Rudolf Oehler and his student Vanessa Schimek (Medical University of Vienna, Austria) described the clearance of apoptotic cells by neutrophils and its potential role in tissue regeneration. Lukas Kenner (Medical University of Vienna, Austria) talked about the role of PDGFRB in anaplastic large cell lymphoma. His student, Tobias Suske, showed that a hotspot gain-of-function mutation in Stat5B occurs in patients with aggressive T cell neoplasia. Karin Nowikovsky (Medical University of Vienna, Austria) investigated the off-target effects of a fatty acid synthase inhibitor in a breast cancer mouse model, identifying a novel synthetic lethality. Julijan Kabiljo, Bergmann group, showed that antibody-dependent phagocytosis induced by trastuzumab could be enhanced by HDAC inhibitors in ex vivo cultures. Balazs Hegedus (University of Duisburg-Essen, Germany) demonstrated how pleural effusions from patients with thoracic malignancies are a source of novel biomarkers to yield new preclinical models to study development of resistance against targeted therapies.

